# Supplementary material for: Stability of eye movement-related eardrum oscillations to acoustic and gravitational/postural manipulations
Source: PLoS One. 2026 Nov 4;21(11):e0361776. doi: 10.1371/journal.pone.0361776 (PMC13734604; doi:10.1371/journal.pone.0361776)
Supplement: Supplementary file 1 [file pone.0361776.s001.docx]

RESPONSE LETTER (PONE-D-26-19909)

General response: We would like to thank the reviewers and the editor for their time and dedication to read, analyze and comment on this manuscript. We have been able to address all points by revising the text and revising the data analysis.

Most importantly, we completely revisited and clarified the inclusion criteria for individual participants, which are now based on purely objective criteria. In doing so, we now base the analysis on a larger participant sample compared to the previous manuscript and report data from n=39 participants for each of the two experiments. These reflect 90% of the n=43 complete datasets available. Although the individual numbers changed, the core results and main conclusions remain the same.

In accordance with the journal’s requirements, the data and scripts used for the analysis are publicly available at GitLab and the Bielefeld University’s publications repository. This information has been included in the “Materials and Methods” section of the manuscript, lines 270–274.

We reordered the Results, moving the text and figure on “Analysis of saccade properties” to the end of the results section, which changed the order of the last three figures. The figures are now:

Fig 1. Schematic representation of the experimental paradigms, not changed.

Fig 2. Suppression of TEOAEs, not changed.

Fig 3. EMREOs for horizontal saccades in silence and with contralateral suppression, not changed.

Fig 4. EMREOs in the head-tilt task, previously Fig. 5.

Fig 5. Regression analysis of EMREOs for the Head tilt task, previously Fig. 6.

Fig 6. Saccade properties, previously Fig 4.

Due to the reorganisation of the text, the references have been reordered, with new references added in accordance with the reviewers’ suggestions. The new numbering is indicated in the marked-up version of the manuscript.

Finally, we changed the title to “Stability of eye movement-related eardrum oscillations to acoustic and gravitational/postural manipulations”.

**Reviewer #1:**

Just a couple of years ago, Groh et al. found that, associated with each horizontal saccadic movement, there was a tympanic movement associated both temporally and in amplitude. To date, the mechanisms involved and their contributions to auditory perception remain unknown. Several studies have been published exploring the possible modulations of these entanglements. This paper reports that two potential modulatory mechanisms in these embryos do not modulate either their amplitude or their temporal course.

This manuscript is very well written, and the terms are very well defined, with the aim of comparing how two known modulators of these elements affect the EMREO time course. Across both experiments, their data revealed no clear modulation of the EMREO time course by these experimental manipulations. This work contributes to elucidating the mechanisms involved by ruling out potential modulators.

While the manuscript is complete and the results are robust. It could be improved by addressing the following comments:

1) The quality of the figures does not enable proper evaluation .- Particularly fig. 4. Colored labels can not be read, although it can be appreciated that there are no differences.

Reply: Figures have been revised and we assured that they were properly exported and saved in high resolution. For Fig 4 (now Fig 6), error shades and traces were improved to avoid misinterpretations, and the labels were positioned differently to enable a better readability. Font sizes were adjusted where appropriate.

2) It is interesting to consider that OHCs may contribute to the EMREOs. However, otoacoustic emissions are associated with a tympanic membrane excursion whose amplitude is much smaller than that presented by the EMREOs, so there is a discrepancy regarding the possible contributions of these cells.

Reply: We agree that the strength of signals generated by the OHCs alone would not be enough to create a signal of the magnitude of the EMREOs. Likely, as discussed in our previous publication (Sotero Silva, Kayser and Bröhl, Hearing Research, 2025), the MEMs are a more likely source to produce the observed oscillations in the eardrum. However, the hypothesis of OHCs as the main generator has been repeatedly discussed in previous studies (Gruters et al., 2018; Lovich et al., 2023), and without clear evidence, certainly cannot be ruled out. Hence, we considered it important to probe a potential contribution of OCHs by starting from a known modulator of the OHCs activity, the MOCR suppression effect. We discuss the likely predominance of MEMs in contributing to the EMREOs (L.667-674) and we added a brief statement to the introduction that typical otoacoustic emissions are smaller than EMREOs (L.69-70).

3) The suppression that can be seen in the reduction of transient evoked otoacoustic emissions (Figure 2) is also associated with high frequencies, in contrast to the frequency of EMREOs, which is around 30 hertz. It might be important to clarify how suppression at such a high frequency could affect tympanic membrane movement in this very low range.

Reply: Indeed, TEOAEs are typically observed at frequencies above 500Hz. This “signal” can be seen in the time course of in-ear recordings and frequency spectra. Importantly, this signal reflects neural and cellular processes that generate (among other time scales) activity in this > 500Hz range. However, the very same processes can be modulated (e.g. strengthened or weakened) at a much slower time scale. Hence, strengthening or weakening of this process could be seen at one time scale (e.g. 20-40Hz, as the EMREO), while another product of this process is seen at a higher time scale (>500 Hz). We have added this reasoning to the discussion (L. 646), although we note that this is speculative. Ultimately, some form of cross-frequency coupling analysis could be performed to understand whether the same causal signal component gives rise to activity at 500Hz as seen in the TEOAE and the much slower EMREO. However, such an analysis is clearly beyond the scope of the present study. Alternatively, it could simply be that TEOAEs and EMREOs are generated by two very different elements in the ear (OHCs and MEMs) and hence emerge at different time scales.

4) Lines 48–50 ("The EMREOs are likely generated... promoting the middle ear muscles...") are slightly complex. The word "promoting" isn't quite the right fit here.

Reply: The sentence was modified to: “The EMREOs are likely generated by motor components in the middle ear and the cochlea. Particularly the middle ear muscles (MEMs) and the outer hair cells (OHCs) have been indicated as key candidates” (L.49-51).

5) Line 67 mentions "one could expect that experimental manipulations... may also affect the EMREO." This is your hypothesis. It would be stronger if stated more explicitly as a prediction: "We hypothesized that if OHCs contribute to the EMREO signal, then manipulations interfering with OHC function, such as the MOCR, would predictably alter the EMREO waveform."

Reply: We state this hypothesis at the end of the same paragraph (L.80-84), which we modified to: “We here tested whether inducing the MOCR, as confirmed by a suppression of contralateral TEOAEs, alters the time course of the EMREO induced by horizontal saccades in the opposing ear. Our assumption is that if OHCs contribute to generating the EMREO, any process that impacts OHCs activity (e.g. the inhibitory effect posed by MOCR) should also alter the EMREO waveform in a predictable way.”

6) Clarification on Head Tilt: It isn't entirely clear if you are investigating the static vestibular signal (the head's orientation relative to gravity) or the proprioceptive signal from the neck muscles. A brief clarification here would help the reader understand which "context" is being tested.

Reply: Our main focus with this manipulation were vestibular processes that are known to modulate eye movements in response to changes in head orientation relative to the body and/or the visual scene. However, indeed our manipulation induces a broad range vestibular and sensory signals, including static vestibular signals, but also proprioceptive feedback and also conceptual knowledge about one’s own head position. We understand that those cannot be properly separated, and cite briefly the contribution of neck signals in the discussion (L.685-687). We also clarified the combined vestibular/proprioceptive nature of the manipulation at multiple instances of the manuscript.

7) Line 111: "ear under study." Use "ipsilateral ear" for more precise anatomical terminology, especially since you are discussing contralateral noise.

Reply: the text was changed accordingly.

8) Head-Tilt Mechanics (Lines 234–241): You mention the eye tracker and chinrest were on a tiltable platform. This is a major strength of your setup as it maintains the geometric relationship between the eye and the camera. However, you should explicitly state if the screen remained stationary (upright) while the participant tilted. Line 240 suggests it did ("stimulus projection was adjusted"). This confirms you are testing a "world-oblique" vs "head-cardinal" conflict, which is a great experimental control.

Reply: Yes, the targets’ projection on the screen was kept upright also for the tilted conditions and the adjustment referred to in the text is related to the position of the targets on the screen, that were slightly lowered and lateralized, but not rotated. This is illustrated in Fig 1 panel B, and the mismatch generated by rotating the head but not the targets is illustrated in Fig 1C. The text was modified to “the stimulus projection was adjusted on the screen (lowered and moved to the side of the tilt) to ensure that the center fixation was at the same level to participants' eyes in each block, but the targets’ locations were kept upright for all conditions” (L.259-262).

9) Explicitly state that the lack of effect from head-tilts suggests the EMREO is craniocentric (fixed to the head’s frame of reference) rather than allocentric or gravity-centered. This supports your conclusion that it might not be a sophisticated spatial "calculator," but rather a simpler motor-sensory "handshake."

Reply: We inserted this point in the Discussion, L.692-698: “Our data support that EMREOs are robust to such an influence of vestibular and proprioceptive signals despite vestibular-ocular reflexes likely operating during the head tilt. The absence of a head tilt effect on the EMREO waveform indicates that the encoding of saccades parametric information is fixed to the head reference, rather than to the body or the visual scene. This interpretation does not support the idea that EMREOs would provide retinocentric spatial information for an adjustment of the computation of sound position.”

**Reviewer #2:**

Summary

This paper considers the question of whether context affects EMREOs and evaluates two particular possibilities: suppression by sound and changes in **postural position**. The first experiment (suppression by sound) builds on known paradigms in which delivery of sound to one ear suppresses otoacoustic emissions in the other ear (OHC suppression via the MOCR). The authors postulate that if EMREOs are caused by a similar mechanism, then a similar pattern of suppression as is seen with OAEs should be observed. The second experiment involves head tilt, with matched rotation of the screen, i.e. permitting comparison of EMREOs in the same head-centered reference frame as a function of two different head postures.

The paper is methodologically sound and the results are interesting. No differences are seen between conditions in either experiment. **This null result adds to the existing literature that the EMREO is robust to the particular circumstances in which the eye movements occur.** The discussion section interpreting the results is well reasoned and nuanced, especially with regard to the implications regarding OHCs vs MEMs – while the contralateral noise may specifically impact the OHCs, the MEMs may also be involved. **The inclusion of cluster-based statistical tests is well motivated and constitutes a notable improvement over previous studies in this field.**

The most important suggestion for improvement is to better document the inclusion/exclusion criteria for subjects, as about 1/3 of the originally tested participants were excluded from the final dataset, apparently based on visual inspection of the data.

Reply: Unfortunately we did not clearly report the selection and inclusion of participants in the previous version. In fact, out of the 47 recruited participants, we only collected EMREO data from n = 43. From these we excluded a number of participants based on criteria that we now clearly describe (see below). We now report the data from n = 39 participants for each task, representing a larger participant sample than analyzed in the previous version and reflecting about 90% of the complete datasets collected for this study.

One suggestion regarding interpretation would be to note the **possibility that there may be individual differences across subjects, which average out at the population level**. This may be more likely to be a factor for Expt. 1 (the contralateral noise) than for Expt. 2, as it is known that contralateral noise suppression thresholds vary across subjects.

Reply: Indeed, the individual data are variable, both in terms of the EMREO signal and the hearing status. For example, the individual thresholds for contralateral suppression vary and in a previous study we noted a significant correlation between middle ear properties and individual EMREO amplitudes (Sotero Silva et al., Hearing Research, 2025). We followed a similar line of thought here and investigated the correlation between the TEOAE suppression and the EMREO amplitude across all 78 ears (L.469-471) “[...] the correlation between the TEOAE and EMREO condition differences were not significant (rho=0.04, p=0.69)”. To us this suggests that variability in suppression thresholds does not confound with variability in the EMREO. However, this inter-individual variability in auditory function and the difficulty to reliably estimate individual suppression thresholds certainly adds uncertainty to this experimental manipulation, which we acknowledge in the Discussion on the “EMREOs and outer hair cells” session, L.661-671.

General Comments

The methods for evaluating and suppressing TEOAEs are consistent with previous research and the within participant analysis is innovative for this type of experiment. Methodologic approaches are well-described, the statistical analyses appear sound, and the findings add to the growing body of knowledge related to EMREOs.

For consideration: **The paper jumps back and forth between two experiments that are not substantively related to each other.** Although there is value in both experiments and it is reasonable to present them in a single paper, **it may be easier to read if aspects of the Methods and Results were reorganized under Experiment 1 and Experiment 2.**

Reply: We chose not to separate the text under Experiment 1 and Experiment 2 because critical sessions in the Methods apply to both experiments (e.g. the experimental setup, the processing and analysis of saccades and EMREOs), while the Results are already separated under different headings. We revised the text to make the relationship between the text and the experiment in question clearer and to remove any ambiguity.

Please confirm that statement claims in the Introduction are appropriately cited. Some examples are highlighted in the Specific Comments (#2 & #3).

Specific Comments

1. The title is misleading as there are no gravitational manipulations; instead, there is a postural change impacted by gravity. A suggestion for more accurate wording—“…to acoustic and **postural changes**”.

Reply: We revised the wording at multiple instances of the text to clarify the combined vestibular and proprioceptive nature of this postural manipulation. We changed the title to “Stability of Eye Movement-Related Eardrum Oscillations to acoustic and gravitational/postural manipulations”.

2. (Introduction, manuscript lines 63-66, “Previous studies suggest that the OHCs may also receive information about oculomotor behavior, possibly as descending information from the thalamus and/or superior colliculus via the superior olivary complex [5,17].”)

The references provided here are from human experiments and though these papers do reference electrophysiologic experiments to support claims of efferent influence of oculomotor signals in the inferior and superior colliculus, it would be best to directly reference such findings. Here are some suggested citations documenting connections from SC to IC to SOC:

From SC to IC:

Coleman, J. R. and W. J. Clerici (1987). "Sources of projections to subdivisions of the inferior colliculus in the rat." J Comp Neurol 262(2): 215-226.

Sparks, D. L. and R. Hartwich-Young (1989). The deep layers of the superior colliculus. The neurobiology of saccadic eye movements. R. H. Wurtz and M. E. Goldberg. New York, Elsevier: 213-255.

From IC to SOC:

Faye-Lund, H. (1986). "Projection from the inferior colliculus to the superior olivary complex in the albino rat." Anat Embryol (Berl) 175(1): 35-52.

There are probably a number of options for references from SOC to cochlea.

Re: the thalamus, which part of the thalamus are you referring to? Can you provide some citations for oculomotor signals in thalamus and a pathway to the periphery?

Reply: We added the indicated references and others. We also decided to not mention the thalamus directly. The text was revised and now reads: “Previous studies suggest that the OHCs may also receive information about oculomotor behavior that can possibly contribute to the generation of the EMREO [5,17]. These signals involve descending information from the superior colliculus via the superior olivary complex, as determined by animal studies [Coleman and Clericei, 1987, Sparks and Hartwich-Young, 1989; Faye-Lund, 1986; Riemann and Reuss, 1998; Suthakar and Ryugo, 1998; Schofield and Coomes, 2006]. However, signals induced by OHC activity, such as otoacoustic emissions, are generally much smaller than typical EMREOs.” (L.65-70).

3. (Introduction, manuscript line 91, “For example, the extraocular muscles…)

Generally, it would be useful to describe and reference the postulated pathway for these kinds of statements.

Reply: We added the pathway related to the head-tilt relevant for the experiment in L97-106. The text now reads: “For example, the extraocular muscles receive feedback from the vestibular system that is taken into account when coordinating eyes and head during gaze control. Moving the head in relation to the body triggers vestibular reflexes and activates eye muscles differently (Higgins et al., 2023; Hikosaka and Maeda, 1973; Morimoto et al., 2011). Frontal-plane head tilts provoke excitatory stimulation in the semicircular canals of the ipsilateral ear and inhibitory stimulation in the contralateral ear. The signal transmitted by the vestibular nuclei leads to activation of the oculomotor and trochlear nerves, generating contraction of the superior rectus and oblique muscles in the ipsilateral eye, and inferior rectus and oblique muscles in the contralateral eye, rotating the eyes opposite to the head orientation (Fife, 2010).”

4. (Methods, manuscript line 161, “an acceleration threshold of 8000°/s”)

Confirming that this would be degrees per second per second?
Reply: We thank you for calling our attention to this. It should be 8000°/s², as it is an acceleration measurement. This was corrected in the text.

5. (Methods, manuscript lines 189-191, “…during the second and third blocks we substituted one of the in-ear probes with the unilateral headphone over which we presented the same suppression noise”)

What was the purpose for two different delivery methods of suppressing noise?

Reply: The previous sentence was somewhat misleading and we clarified the description. For the TEOAEs, the noise was delivered unilaterally by the in-ear probes. Text in L.222-223 was changed to “For the latter, during the second and third blocks we presented the clicks ipsilaterally and the suppression noise contralaterally with the in-ear probes.”

However, we used another headphone to induce contralateral suppression because we disconnected the sound-input of the ER-10C system during the EMREO paradigm to ensure that no sounds possibly produced by the computer or the audio-amplifier would be sent to the ear from which we recorded the EMREOs. For that reason the noise was delivered by the unilateral headphone and adjusted the appropriate intensity using the sound-level meter. The text in the subsession “Measurement and suppression of EMREOs” was changed in L.241-243 to “For this, we substituted one of the in-ear probes with the unilateral headphone over which we presented the noise, while the contralateral ear was kept with the in-ear probe for EMREO recording (Fig. 1A).”

We now also anticipate this matter in the “Experimental Setup” session, L.174-176 “While recording the EMREO from a given year, the respective acoustic input to that ear was disconnected from the audio-amplifier to avoid any potential contamination of the recorded EMREO signal.”

6. (Methods, manuscript line 200)

Typographical error: ‘steadily’ instead of ‘steady’.

Reply: The text was changed accordingly.

7. (Methods, manuscript lines 215-217)

There are 240 saccades per block. How many blocks are presented in total?

Reply: For each participant we recorded 720 trials for Experiment 2. Text in L.252 was changed to “resulting in a total of 240 saccades per block, leading to 720 trials in total”.

8. (Methods, manuscript lines 234-235, “The main rationale of this experiment was to test whether tilting the head would influence the EMREO time course.”)

What was the rationale for expecting a change in EMREO time course? This is an interesting and unexpected hypothesis.

Reply: Our rationale was that any change in the continuous feedback from the vestibular system and about body posture would affect oculomotor control and therefore possibly add or change those signals about oculomotor behavior that are relayed to the ear and which induce the EMREO. Both the source and the nature of the signals carried to the ear inducing EMREOs are unknown, and deemed it interesting and important whether these are sensitivity to vestibular state and body posture.

9. (Methods, manuscript line 247)

Typographical error: ‘misaligns’ instead of ‘misaligned’.

Reply: The text was changed accordingly.

10. (Methods, manuscript line 267, “(see below)”)

Are you referring to text or a figure? Please specify what ‘below’ means?

Reply: We refer to text, specifically the session “Analysis of saccade characteristics”. Text was changed accordingly. In other sentences with a similar structure we ensured to indicate which session is referred).

11. (Methods, manuscript lines 271-276, “Analysis of saccade characteristics”)

It is unclear from the text if this analysis applies only to Experiment 2 but the reference is for Figure 5. Here is an example of where jumping back and forth between the two experiments can become confusing. If you choose to keep the basic structure of the methods section, please make sure you clarify any ambiguous text.

Reply: This mistake must have arisen when changing the order of figures while preparing the manuscript. Fig 6 (previously Fig 4) should be cited here. The text was changed to “To ensure that the saccades driving the EMREOs in the different experimental conditions are comparable in their characteristics (silence and noise in Experiment 1, different head tilts in Experiment 2) [...] and compared the resulting averages between conditions for both experiments across participants (Fig. 6, panel A for Experiment 1, panels B and C for Experiment 2)”. (L. 296-301).

12. (Methods, manuscript lines 290-292, “We then visually inspected the resulting epoch-averaged EMREO time courses and excluded participants with noisy or flat EMREOs from subsequent analysis. Based on these criteria we retained data from 31 participants for each task.”)

The authors describe objective exclusion criteria (lines 281-289) but then appear to use a subjective method of exclusion via visual examination and interpretation. Is that correct? It is concerning that approximately 1/3 of the original data set was eliminated (31 of 47 subjects retained for Experiment 1 and 29 of 47 for Experiment 2). This has not been the case in most previously published EMREO research. Can the authors elaborate as to why they chose to exclude such a large percentage of the original subject pool through visualization of the signal? If possible, it would be better to provide some more objective criteria for the basis of the exclusions.

Reply: We agree that the previous description was very incomplete and in part also misleading. We revised the methods to be precise about the criteria. First, while recruiting n = 47 participants for the study, only n = 43 proceeded to the actual EMREO recordings as some participants were excluded based on their hearing thresholds or failure of the eye tracking system to track their eyes (now described in L.162-165). Then, the data of 4 participants for one of the experiments (suppression) was incomplete and these were not included in the analysis of that experiment but for the analysis of the head-tilt manipulation (L.303-306). This leaves n = 39 complete, good datasets for Experiment 1, and n = 43 for Experiment 2. In the previous manuscript the exclusion of those was somewhat loosely summarized as “based on visual inspection”.

For these (n = 39/43) participants we calculated the EMREOs and inspected these visually, and using objective criteria. First, we calculated a signal to noise score (L.319-322) and excluded participants for which the average EMREO did not exhibit a maximal deflection with SNR > 1.5. This objective criterion aligns well with visual inspection of what would be considered an “obvious EMREO”. In addition, the EMREO of some participants was visually noisy, possibly because on a subset of saccades the signal exceeded the AD limit of the system (based on pure visual inspection). Based on this we formulated a criterion based on which we excluded participants with a peak EMREO of > 20mV (L.322-323). Based on these two criteria we did not exclude any participant for Experiment 1, hence reporting data from n = 39 for the suppression experiment. We did however exclude data from 4 participants for Experiment 2, hence also reporting n = 39 data for the head-tilt manipulation. For one specific analysis (the condition-averaged EMREO in Experiment 2 we had to omit 2 further participants as they presented to few saccades in one of the directions, L.352), however, these two participants were included in the regression analysis for this experiment.

13. (Results, Figure 2)

Consider combining the no-noise and noise-suppressed data onto the same graphs for easier comparison.

Reply: We appreciate the suggestion, but considering that panel B shows the suppression value, not the TEOAE amplitude for the suppression blocks, we believe that collapsing the panels into one could be misleading. We ensured that the figure legends clearly describe what is shown where.

14. (Results, manuscript lines 409-410, “We then tested whether the EMREO time course is altered by the presence of a contralateral noise”)

Is the question about changes in latency of the peaks/troughs of the waveform, about the amplitude of the signal, or both? This also relates to Specific Comment #8. Elaborating on the hypothesis would clarify this point.

Reply: The time course of EMREOs that we compare effectively comprises both amplitude and waveform. Any (significant) difference in time course could in principle result from a mere change in amplitude, a change (e.g., shift) in EMREO latency, or a combination. We clarified the text to clearly mention this.

15. (Results, Figure 4)

Recommend splitting this into 2 figures—4A (OAE suppression results) as Figure 4, but the remaining panels concerning the head tilt experiment as a separate figure, to be placed in the manuscript after the results from that experiment.

Reply: Although we decided not to split the figure, we entirely moved the “Analysis of saccade properties” session to the end of the Results. This way the readers can focus on the main Results (TEOAE and EMREO) without a “break” and later appreciate the information from the saccades in both Experiments, which works as a complement that increases the reliability of the conditions’ comparisons. Because of that, the order of the last three figures is changed as Fig 4 “Saccade properties” becomes Fig 6. The new order of Figures is explained in the general response.

16. Interpretation of Expt. 2 and the underlying oculomotor commands: head tilt induces a modest torsional counter-rotation in humans, so the oculomotor commands will not have been exactly the same across the three different postural conditions. Given the EMREO results did not differ, this is a minor comment but may be useful for providing motivation for the experiment.

Reply: we included an expanded description of this effect in the Introduction, in L.99-106.

17. (Conclusion, manuscript line 653)

[Reference 66] It is possible that the intent was to reference a different BioRxiv paper from the same research group: King and Groh, Saccade-related sound pulses and phase-resetting contribute to eye movement-related eardrum oscillations (EMREOs).

Reply: In fact, we cited the wrong paper. The reference was corrected (now reference 70).

We are signing this review because we prefer to be transparent with the authors regarding our feedback.

Sincerely,

Cynthia King and Jennifer Groh
