# Supplementary material for: Stability of eye movement-related eardrum oscillations to acoustic and gravitational/postural manipulations
Source: PLoS One. 2026 Nov 4;21(11):e0361776. doi: 10.1371/journal.pone.0361776 (PMC13734604; doi:10.1371/journal.pone.0361776)
Supplement: Supplementary file 2 [file pone.0361776.s002.docx]

RESPONSE LETTER (PONE-D-26-19909R1)

General Response: We would like to thank the reviewers for the time dedicated to review our manuscript. We addressed all the comments by Reviewer 2. This led to the removal of panel C in Figure 1 and adjustments in panel B and the legend. The order was not changed. Text changes are pointed in the response below.

Review Comments to the Author
**Reviewer #1:** No comments. They have properly replied to all my queries. Therefore, I suggest its acceptance in PLOS ONE

**Reviewer #2:** Thank you for addressing the feedback from both reviewers. Multiple aspects of the manuscript are now clearer. However, some problems remain, and some new problems were introduced (or newly recognized). It is critical these problems be addressed before the manuscript can be published.

1. Abstract (new problem): The closing sentence reads “Together with other recent studies these finding advocate for a stability of the EMREO time course towards multiple experimental modulations and fuel speculations that the signal may serve as a temporal reference frame rather than a spatial coordinate transform function when combining signals across the senses.”
This sentence has two problems. First, the more important problem is the phrase “rather than a spatial coordinate transform function”. We think the authors have misunderstood the findings of their tilt experiment. In their response to reviewer 1, they state “The absence of a head tilt effect on the EMREO waveform indicates that the encoding of saccades parametric information is fixed to the head reference, rather than to the body or the visual scene. This interpretation does not support the idea that EMREOs would provide retinocentric spatial information for an adjustment of the computation of sound position.” This last sentence is incorrect: what is needed for a spatial coordinate transform connecting head-centered auditory input with eye centered visual input is a signal of eye position with respect to the head. That is exactly what the authors observed in the tilt experiment, because tilting the head had no effect. Accordingly, the final sentence of the abstract should be revised. We recommend flipping it around to state that the tilt experiment supports the spatial coordinate transform role, but does not eliminate a role in other possibilities such as temporal synchronization with visual input. Other places in the manuscript need to be updated as well (see below for comments re: the Discussion).

Reply: The text was changed accordingly in the abstract (L.34-37),were it now reads:  “We discuss how this fuels speculations that the signal may serve as a temporal reference frame, while also being compatible with the notion that the EMREO reflects a spatial coordinate transform for combining signals across the senses”. We also modified the Discussion (L.694-696, L.698-702), see reply to comment number 5.

Second, the phrase “EMREO time course” – this phrase is overly specific. There is nothing in the manuscript that specifically singles out any temporal properties of the EMREO as opposed to its overall waveform. The phrase “time course” can be removed and just say “EMREO”.

Reply: We removed the term “time course” in this and other instances along the text.

2. Tilt experiment design (newly recognized problem): The low resolution of the figure as originally presented prevented us from realizing that the target display was not in fact rotated, so the set of saccades as defined with respect to the head were not the same in the two tilt conditions. It is unfortunate that the target angles were every 45 degrees whereas the head tilt was 30 degrees, so there is no direct comparison of the exact same head-centered saccades in the tilted vs upright condition. The analyses handles this by using regression analysis, but it does weaken the conclusions and the reasons for this design choice and the resulting limitations should be made clear. If it was accidental or stemmed from a miscommunication between authors, that’s ok – not everything in life goes according to plan – but it would be good to acknowledge the limitations of relying on regression to work around this design choice.

Reply: We don’t see the regression analysis as a weakness of our interpretations. First, the regression analysis is complementary to the standard statistical assessment performed in the EMREO data. Second, many previous studies on the field, from different groups, applied regression models, even as the main or sole analysis. We now state this more explicitly in the methods and the discussion. By confirming the null result across two analyses we feel even more confident about this.

The reason we missed this in the original manuscript stems from both the figure graphics and the text description. Suggestions for improvement below:
a) Improve the visualization of how the targets shift relative to the head in Figure 1B. The task figure for the tilt experiment does not make clear that the targets were not rotated to match the head tilt. The problem is that all the targets are white. You could color code each of the targets so that it is more apparent that the targets did not rotate with the head tilt but rather simply translate to accommodate the sideways translation of the head when tilted. Something similar to the color-coding used in Figure 6D could work well.

Reply: We adjusted Figure 1, adding the suggested color scheme in panel B to make the visualization of the target's orientation easier. We removed panel C to avoid misinterpretation. It will not be missed since we improved the textual description of the targets (see below). We also added in L. 254-256 “Our initial aim was to rotate the platform by the same degree as the (circularly-symmetrical distributed saccade targets; 45°) but this was not feasible, hence the reduced tilt for this manipulation.”

b) The description of the head tilt relative to the targets is somewhat difficult to unpack:
Lines 259-262— For the head-tilted conditions, the stimulus projection was adjusted on the screen (lowered and moved to the side of the tilt) to ensure that the center fixation was at the same level to participants' eyes in each block, but the targets’ locations were kept upright for all conditions (see Fig 1B). Suggested revision: The words “translate” and “rotate” would be more clear here, e.g. “For the head-tilted conditions, the stimulus projection was translated on the screen so that center fixation was at a consistent position relative to the participants’ eyes in each block (lowered and moved to the side of the tilt), but the target array was not rotated but remained upright in its original orientation (see Fig 1B).

Reply: We added this formulation (L.258-261), which now reads: “For the head-tilted conditions, the stimulus projection was translated on the screen so that center fixation was at a consistent position relative to the participants’ eyes in each block (lowered and moved to the side of the tilt), but the target array was not rotated but remained upright in its original orientation (see Fig 1B).”

Lines 263-267— Because in the tilted blocks the axis of the head and the calibration grid on the screen were rotated relative to each other, we transformed all eye positions into head-centered coordinates for data analysis. Note that although the degree of head tilt (30°) was not the same as the spacing of saccade directions (45°), this difference does not constrain the interpretation of the results (see below in the Results session, also Fig 1C).
Concern: The phrase “this difference does not constrain the interpretation of the results” – this is a rather strong claim; it depends on regression being a good model and that all the relevant factors interact linearly (horizontal, vertical). This should be watered down, here and any other places it is discussed.

Reply: We changed the text to make it more specific that we refer to the regression analysis (L.266), it now reads “this difference does not constrain the interpretation of the regression analysis.”

3. When z-scoring to screen for outliers, did you intend to say that trials were excluded if they exceed 4.5 SD? Or is this comparing the EMREO time window to the remainder (or the entire) recording epoch as a criteria for determining the presence of a signal?

Lines 313-315— The resulting data epochs were z-scored to screen for extreme values: epochs for which the maximal z-score of the EMREO signal at any point in time exceeded 4.5 SDs of the overall signal were included in the epoch average.

Reply: The text in L.314 was changed to “epochs for which the maximal z-score of the EMREO signal at any point in time exceeded z=4.5 were excluded from further analysis.”

4. Line 174 —Typographical error: should be ear, not year

Reply: We thank you for calling our attention to this mistake. It was corrected.

5. Line 692-694 —“Our data support that EMREOs are robust to such an influence of vestibular and proprioceptive signals despite vestibular-ocular reflexes likely operating during the head tilt”
This is strange statement. The VOR occurs during head movement, not during a static head position. The subjects were presumably fixating stably. This should be rephrased. Perhaps what is meant is that there is, generally speaking, a difference in the vestibular signals reaching the brain in the tilted vs. upright conditions.

Reply: The text was changed to (L.693-702): “Our data support that EMREOs are robust to such an influence of vestibular and proprioceptive signals despite a difference in the vestibular signals reaching the brain in the tilted vs. upright conditions. The absence of a head tilt effect on the EMREO waveform suggests that this signal is reflecting saccade information in head coordinates, rather than to the body or the visual scene. Thereby this signal may reflect information about eye movements in head coordinates that somehow induced in the processing of acoustic information received in head coordinates. Whether and how this could facilitate the spatial alignment of head and eye specific auditory and visual signals, as speculated previously, still needs to be understood better.”

6. Line 696-698 —“This interpretation does not support the idea that EMREOs would provide retinocentric spatial information for an adjustment of the computation of sound position.”
This is incorrect. Rather, the tilt experiment suggests that the EMREO encodes information about eye movements relative to the head, which is indeed the signal needed for a spatial coordinate transformation. The tilt experiment does not test whether there is eye-centered information in the EMREO signal (but Lovich et al PNAS 2023 already tested that possibility and demonstrated that there is both eye-centered and head-centered information in the EMREO).

Reply: The statement mentioned was removed from the text.
